# I Know My Neighbour: Individual Recognition in *Octopus vulgaris*

**DOI:** 10.1371/journal.pone.0018710

**Published:** 2011-04-13

**Authors:** Elena Tricarico, Luciana Borrelli, Francesca Gherardi, Graziano Fiorito

**Affiliations:** 1 Dipartimento di Biologia Evoluzionistica “Leo Pardi”, Università degli Studi di Firenze, Firenze, Italy; 2 Telethon Institute of Genetics and Medicine, Napoli, Italy; 3 Stazione Zoologica “Anton Dohrn”, Napoli, Italy; University of Alabama, United States of America

## Abstract

**Background:**

Little is known about individual recognition (IR) in octopuses, although they have been abundantly studied for their sophisticated behaviour and learning capacities. Indeed, the ability of octopuses to recognise conspecifics is suggested by a number of clues emerging from both laboratory studies (where they appear to form and maintain dominance hierarchies) and field observations (octopuses of neighbouring dens display little agonism between each other). To fill this gap in knowledge, we investigated the behaviour of 24 size-matched pairs of *Octopus vulgaris* in laboratory conditions.

**Methodology/Principal Findings:**

The experimental design was composed of 3 phases: Phase 1 (acclimatization): 12 “sight-allowed” (and 12 “isolated”) pairs were maintained for 3 days in contiguous tanks separated by a transparent (and opaque) partition to allow (and block) the vision of the conspecific; Phase 2 (cohabitation): members of each pair (both sight-allowed and isolated) were transferred into an experimental tank and were allowed to interact for 15 min every day for 3 consecutive days; Phase 3 (test): each pair (both sight-allowed and isolated) was subject to a switch of an octopus to form pairs composed of either familiar (“sham switches”) or unfamiliar conspecifics (“real switches”). Longer latencies (i.e. the time elapsed from the first interaction) and fewer physical contacts in the familiar pairs as opposed to the unfamiliar pairs were used as proxies for recognition.

**Conclusions:**

Octopuses appear able to recognise conspecifics and to remember the individual previously met for at least one day. To the best of our knowledge, this is the first experimental study showing the occurrence of a form of IR in cephalopods. Future studies should clarify whether this is a “true” IR.

## Introduction

Individual recognition (IR) is regarded as to be an important prerequisite for the evolution of a wide range of social behaviours, from mate choice and parental care to territorial defence and dominance hierarchies. The intrinsic complexity of the phenomenon, along with the wide diversity of its expression across the animal kingdom, has generated a debate on the defining features that make up the process [1]–[8]. The dichotomy between “true” IR and “binary” or “class-level” IR has been proposed. In true IR, the receiver learns the individual-distinctive features of the signaller and associates these characteristics with individual-specific information about it [9], [10]. Apparently, this is a sophisticated task that requires specific perceptual and discrimination abilities by the receiver to identify a “unique set of cues” [9] emitted by the signaller [11], [12]. As a consequence, such ability has been assigned to taxa characterized by complex nervous systems or cognitive adaptations, such as fish, birds, and mammals (reviewed in [10]). Recent studies, however, have extended its occurrence to some invertebrate species (insects [13], [14] and decapod crustaceans [15]–[19]; reviewed in [10]). Indeed, research in invertebrates has been hampered by the scarcity of experimental tests capable of discriminating between true IR and other forms of recognition [10]. The majority of studies conducted thus far has been able, at the best, to document the existence in invertebrates of a second form of IR, the binary or class-level IR. In this case, the receiver classifies the signaller into heterogeneous subgroups, such as familiar/unfamiliar or dominant/subordinate [1], [20]–[22]. However, as pointed out by Barnard & Burk [3], a strict distinction between true and binary IR appears fallacious if the ability to recognise conspecifics is regarded as a skill that acts on a gradient of “cue complexity ranging from simple cues to complexes possibly beyond the level of the individual” (pg. 66). Similarly, Steiger & Müller [7] suggest a less restrictive definition of IR, where class-level recognition should be viewed as a form of IR.

Among invertebrates, octopuses are an ideal model organism to explore IR for several reasons. Octopuses exhibit highly sophisticated behaviours and complex learning capabilities together with a well-developed central nervous system with intriguing analogies to the mammalian brain [23] (reviewed in [24]). A well refined neuronal organization [24], [25] is the “hardware” regulating their vertebrate-like behavioural machinery that mirrors unusual cognitive abilities for an invertebrate, such as the use of tools for defence [26] (reviewed in [27], [28]), communication with visual cues [29], personality [30], problem solving and social learning [31]–[33], point-to-point arm movements [34], [35] and long-term memory in both visual and tactile tasks (reviewed in [24], [36]). Octopuses show different temperaments, have eye and arm preferences, play, and recognise their caretakers in the laboratory [37]–[39]. Despite the plethora of information documenting their extremely rich behavioural repertoire (reviewed in [24], [40]), knowledge of the social behaviour of octopuses (and of cephalopods in general) is as yet scanty. The existence of IR in cephalopods has been only hypothesized whereas dedicated studies on the issue are still absent (for exceptions see [21]; reviewed in [29]).

Contrary to squids that are known to form fish-like schools (reviewed in [29]), octopuses are typically regarded as solitary animals (reviewed in [29], except *Eledone moschata* [41]), although some species may live in high densities (e.g. *Octopus joubini*, *O. briareus*, *O. bimaculoides* reviewed in [29]). The paucity and simplicity of their reciprocal interactions, such as avoidance or physical contact, have led researchers to categorize octopuses as “asocial” ([42] and reviewed in [29]). There are, however, a number of clues in favour of the octopuses' ability for IR. Octopuses produce a large number of body patterns that are not only used as defence systems (e.g. camouflage [43]) but also as an intraspecific means of communication, particularly in the contexts of fighting and mating (reviewed in [40], [44]). In natural conditions, some species are considered territorial *sensu* [45]: they occupy home dens for days or weeks and defend them from conspecifics. Usually, the area around a den is not defended (*Abdopus aculeatus* [46], *O. briareus*, *O. cyanea, O. bimaculoides, O. dofleini* reviewed in [29]), but the inhabitants of neighbouring dens only seldom interact between each other [47]. This is likely the expression of the “dear enemy” phenomenon, which explains the reduced aggression between neighbours in territorial animals [48]. A similar phenomenon has also been described in crabs, stomatopods, frogs, lizards, fish, birds, and mammals (see [10], [49]). Individuals of distant areas (the “strangers”) may be regarded by a given animal to be potentially more dangerous than individuals from neighbouring areas (the “dear enemies”) because they are more likely in search of a new territory [50]. On the contrary, the relatively peaceful coexistence between neighbouring individuals is adaptive in that it avoids the costs of frequent fights [49]. As a consequence, IR can be a prerequisite of the dear enemy phenomenon.

Finally, laboratory groups of octopuses have been described to form and maintain dominance hierarchies (*E. moschata, O. bimaculoides, O. cyanea, O. joubini*, *O. maya*, *O. rubescens, O. vulgaris*; reviewed in [29]), although these may be artefacts due to the laboratory setting when compared to their natural territorial behaviour. Several taxa, including decapods, lizards, canaries and cats, may switch from being solitary or territorial in the field to forming dominance hierarchies when they are confined in laboratory groups [51]–[52]. Under similar conditions, octopuses alter their social organization from solitary to hierarchical.

Based on the above premises, we hypothesised here that octopuses are capable of IR in the broad sense (i.e. either true or binary IR) and that they can recognise familiar neighbours. We thus investigated the agonistic behaviour of size-matched pairs of *Octopus vulgaris* (Cuvier, 1797, Mollusca, Cephalopoda) in laboratory conditions. *Octopus vulgaris*, a benthic species distributed worldwide in temperate and tropical waters, is well-known for its complex learning abilities and highly sophisticated nervous system (reviewed in [24]). We designed an experiment composed of three phases (acclimatization, cohabitation, and test) to assess whether this species could recognise a conspecific previously met. Longer latencies (i.e. the time elapsed from the first interaction) and fewer physical contacts were used here as proxies for IR.

## Results

### Phase 1: acclimatization

During Phase 1, all octopuses attacked the offered crab but the latency of attack was longer in sight-allowed rather than in isolated pairs (data not shown). All animals improved their performance over time as the octopuses became more adapted to the experimental setting [53].

### Phase 2 (cohabitation): sight-allowed vs isolated pairs

Overall, female-male and male-male pairs did not differ for any variable among days in both sight-allowed pairs (two-way repeated measures MANOVA: *Λ* pairs = 0.70, df = 7,24, P = 1.30; *Λ* days = 0.80, df = 14,48, P = 0.36; *Λ* days × pairs  =  0.52, df = 14,48, P = 1.16) and isolated pairs (two-way repeated measures MANOVA: *Λ* pairs = 0.95, df = 7,24, P = 0.98; *Λ* days = 0.61, df = 14,48, P = 0.50; *Λ* days × pairs  =  0.65, df = 14,48, P = 0.63). The subsequent univariate analyses confirmed the above results but only for sight-allowed pairs (Table S1). Dominance in isolated pairs significantly increased over time, being higher in female-male rather than in male-male pairs, and the number of physical contacts and ink jets decreased without any difference between types of pair (Table S1).

**Figure 1:**
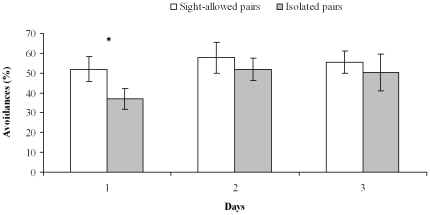
Percentages of avoidance in sight-allowed and isolated pairs. Mean (± SE) percentage of interactions with no physical contacts (avoidance) in sight-allowed (*n* = 12) and isolated (*n* = 12) pairs for each of the three days of cohabitation (Phase 2). *: *P*<0.05.

**Figure 2:**
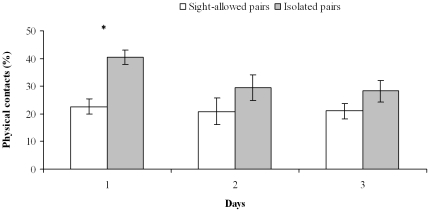
Percentage of physical contacts in sight-allowed and isolated pairs. Mean (± SE) percentage of physical contacts in sight-allowed (*n* = 12) and isolated (*n* = 12) pairs for each of the three days of cohabitation (Phase 2). *: *P*<0.05.

After having merged the data from female-male and male-male pairs, a significant difference was found among days and between sight-allowed and isolated pairs (but not for the interaction days/pairs) (two-way repeated measures MANOVA: *Λ* pairs  =  0.44, df = 16,118, P<0.001; *Λ* days = 0.43, df = 8,59, P<0.001; *Λ* days × pairs  =  0.72, df = 16,118, P = 0.11). Specifically, dominance and the percentage of avoidance increased over time, whereas the number of physical contacts and ink jets decreased (Tables 1, S2–S3; Figs 1–2).

**Table 1:** Comparisons between sight-allowed and isolated pairs for the recorded variables during cohabitation (Phase 2).

	DAYS				PAIRS				DAYS × PAIRS		
	*F*	*df*	*P*	Hierarchy	*F*	*df*	*P*	Hierarchy	*F*	*df*	*P*
Latency of first interaction (s)	1.69	2, 69	0.19	1 = 2 = 3	8.68	1, 70	**0.005**	SP>IP	1.00	2, 66	0.91
Number of interactions	0.29	2, 69	0.75	1 = 2 = 3	1.81	1, 70	0.72	SP = IP	0.72	2, 66	0.49
Length interactions (s)	0.10	2, 69	0.86	1 = 2 = 3	5.68	1, 70	**0.02**	IP>SP	0.48	2, 66	0.62
Number of all behavioural patterns	0.66	2, 69	0.52	1 = 2 = 3	0.84	1, 70	0.35	SP = IP	0.83	2, 66	0.44
Physical contacts (%)	3.50	2, 69	**0.04**	1>2 = 3	3.38	1, 70	**0.04**	IP>SP	1.08	2, 66	0.34
Avoidance (%)	3.37	2, 69	**0.04**	1 = 2 = 3*	3.32	1, 70	**0.04**	SP>IP	1.61	2, 66	0.21
Dominance (%)	5.64	2, 69	**0.002**	2 = 3>1	3.68	1, 70	**0.03**	SP>IP	4.70	2, 66	**0.01**
Number of ink jets	14.32	2, 69	**0.001**	1>2 = 3	3.35	1, 70	**0.04**	IP>SP	3.68	2, 66	**0.04**

Comparisons among the three days of cohabitation (1 = Day 1, 2 = Day 2, 3 = Day 3), and between pairs (sight-allowed: SP, *n* = 12; isolated: IP, *n* = 12) for the recorded variables after a two-way repeated measures MANOVA followed by univariate tests for between-subjects effects (statistic: *F*; factors: days and sight-allowed/isolated pairs), followed by Tukey's HSD. Significant differences are denoted in bold. * means no significant difference after Tukey's HSD.

**Figure 3:**
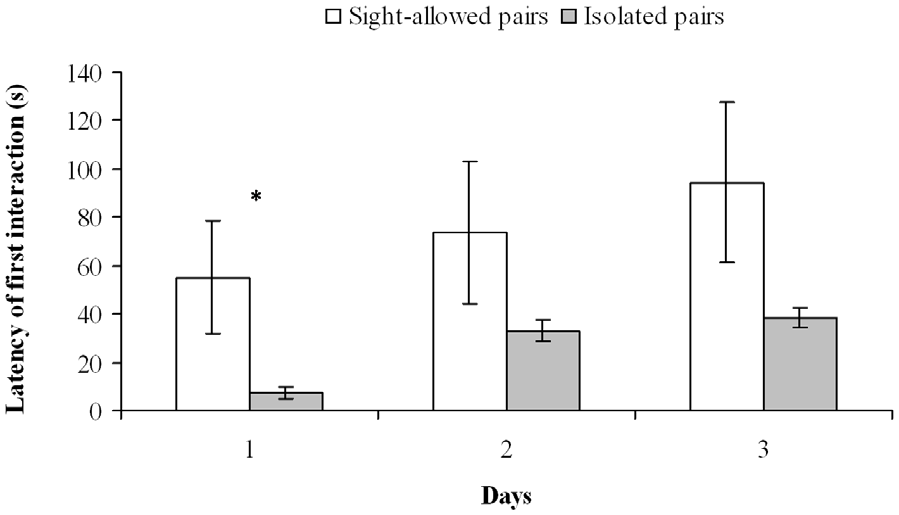
Latency of the first interaction in sight-allowed and isolated pairs. Mean (± SE) latency of first interaction in sight-allowed (*n* = 12) and isolated (*n* = 12) pairs for each of the three days of cohabitation (Phase 2). *: *P*<0.05.

For all variables, sight-allowed and isolated pairs differed at Day 1 (one-way MANOVA: *Λ* = 0.19, df = 8,15, P<0.001), but not at Day 2 (one-way MANOVA: *Λ* = 0.47, df = 8,15, P = 0.10) and Day 3 (one-way MANOVA: *Λ* = 0.48, df = 8,15, P = 0.11) (Table 2, S2). Octopuses of sight-allowed pairs were less prone to interact and more often avoided each other (Tables 1–2, S2–S3; Figs 1,3), whereas octopuses of isolated pairs interacted for longer and executed more numerous physical contacts and ink jets (Tables 1–2, S2–S3; Fig. 2)

**Table 2:** Comparisons between sight-allowed and isolated pairs for each of the three days of cohabitation (Phase 2).

	1^st^ Day			2^nd^ Day			3^rd^ Day		
	*F*	*P*	Hierarchy	*F*	*P*	Hierarchy	*F*	*P*	Hierarchy
Latency of first interaction (s)	5.07	**0.04**	SP>IP	2.30	0.14	SP = IP	3.48	0.08	SP = IP
Number of interactions	0.09	0.93	SP = IP	2.31	0.14	SP = IP	1.10	0.31	SP = IP
Length interactions (s)	5.80	**0.03**	IP>SP	1.81	0.19	SP = IP	0.90	0.33	SP = IP
Number of all behavioural patterns	0.08	0.93	SP = IP	0.02	0.87	SP = IP	1.80	0.19	SP = IP
Physical contacts (%)	25.61	**<0.001**	IP>SP	0.42	0.69	SP = IP	3.70	0.07	SP = IP
Avoidance (%)	15.00	**0.001**	SP>IP	0.65	0.43	SP = IP	0.48	0.50	SP = IP
Dominance (%)	2.25	**0.04**	SP>IP	0.03	0.86	SP = IP	0.04	0.84	SP = IP
Number of ink jets	4.81	**0.04**	IP>SP	2.20	0.15	SP = IP	3.67	0.07	SP = IP

Comparisons for each of the three days of cohabitation between sight-allowed (SP: *n* = 12) and isolated pairs (IP: *n* = 12) for the recorded variables after a one-way MANOVA, followed by univariate tests for between-subjects effects (statistic: *F*). Significant differences are denoted in bold.

### Phase 3 (test): familiar vs unfamiliar pairs

Overall, the different types of pair significantly differed for all variables (two-way MANOVA: *Λ* sight allowed/isolated pairs = 0.41, df = 7,14, P = 0.04; *Λ* familiar/unfamiliar  =  0.15, df = 7,14, P<0.001; *Λ* pairs × pairs  =  0.70, df = 7,14, P = 0.56). Familiar and unfamiliar pairs differed for all the variables analysed except for the number of the behavioural patterns executed (Table 3). In familiar pairs, latency was longer, dominance was significantly higher and octopuses avoided each other more frequently. On the contrary, unfamiliar individuals interacted for longer and more often executed physical contacts (Table 3).

**Table 3:** Comparisons between familiar/unfamiliar pairs and between sight-allowed/isolated pairs for the recorded variables during the test (Phase 3).

	Sight allowed/Isolated pairs				Familiar/Unfamiliar pairs				Pairs × Pairs		
	*F*	*df*	*P*	Hierarchy	*F*	*df*	*P*	Hierarchy	*F*	*df*	*P*
Latency of first interaction (s)	8.90	1, 20	**0.007**	SP>IP	8.90	1, 20	**0.007**	FA>UN	7.49	1, 20	**0.01**
Number of interactions	3.67	1, 20	0.07	SP = IP	8.07	1, 20	**0.01**	FA>UN	1.26	1, 20	0.27
Length interactions (s)	0.07	1, 20	0.80	SP = IP	15.20	1, 20	**0.001**	UN>FA	0.89	1, 20	0.36
Number of all behavioural patterns	2.04	1, 20	0.17	SP = IP	3.49	1, 20	0.08	FA = UN	0.22	1, 20	0.64
Physical contacts (%)	2.07	1, 20	0.17	SP = IP	14.89	1, 20	**0.001**	UN>FA	0.06	1, 20	0.81
Avoidance (%)	6.78	1, 20	**0.02**	SP>IP	17.32	1, 20	**<0.001**	FA>UN	0.38	1, 20	0.54
Dominance (%)	0.06	1, 20	0.81	SP = IP	13.16	1, 20	**0.002**	FA>UN	0.06	1, 20	0.81

Comparisons between familiar (FA, *n* = 12) and unfamiliar (UN, *n* = 12) pairs and between sight-allowed (SP, *n* = 12) and isolated pairs (IP, *n* = 12) for the recorded variables after a two-way measured MANOVA followed by univariate tests for between-subjects effects (statistic: *F*; factors: sight-allowed/isolated pairs and familiar/unfamiliar pairs). Significant differences are denoted in bold.

In familiar pairs, sight-allowed pairs had a longer latency and a higher percentage of avoidance than isolated pairs as shown by both univariate analyses (Table 3) and the one-way MANOVA (*Λ* = 0.17, df = 4,7, P = 0.16; latency: F = 11.12, df = 1,20, P = 0.008; avoidance: F = 5.38, df = 1,20, P = 0.04; other variables: F between 0.17 and 2.13, df = 1,20, P between 0.18 and 0.69; Table S4). On the contrary, in unfamiliar pairs no difference was found between sight-allowed and isolated pairs for all variables (one-way MANOVA: *Λ* = 0.48, df = 4,7, P = 0.73; F between 0.00 and 4.80, df = 1,20, P between 0.07 and 1.00; Table S4).

Dominance hierarchies established were maintained with time in familiar pairs; on the contrary, eight former alphas of unfamiliar pairs became betas and three former alphas remained alphas, while dominance remained undetermined in the last pair. No ink jet was ever recorded in this phase.

## Discussion

This study reports the first experimental evidence of *O. vulgaris*' ability to recognise a familiar conspecific and to remember it for at least one day. As shown during the test phase, unfamiliar pairs, i.e. pairs composed of individuals that have had no previous experience of each other, executed more numerous physical contacts and showed shorter latencies than familiar pairs, being thus more aggressive and prone to interact. Besides, reversals of dominance (i.e. alphas switched to betas and, consequently, betas to alphas) were only observed in unfamiliar pairs. Taken together, these results seem to support our hypothesis that *O. vulgaris* can discriminate familiar from unfamiliar conspecifics, meaning that it is able of, at least, class-level or binary IR *sensu* [10]. To the best of our knowledge, such an ability was never found in other cephalopods. For example, in groups of cuttlefish (*Sepia officinalis*), dominance hierarchies are maintained by the “winner & loser effects”: the behaviour of a cuttlefish is independent of the familiarity or the identity of the opponent but results from its personal experience of wins and losses [21]. Previous studies on octopuses revealed the formation of dominance hierarchies in groups of, for example, *O. bimaculoides* [54], but did not investigate the mechanisms underlying the maintenance of such hierarchies over time. Binary IR should have an adaptive value for *O. vulgaris* being the likely proximate mechanism regulating the “dear enemy phenomenon”. Indeed, the recognition of a familiar neighbour might explain the scarcity of interactions between octopuses, as observed in the field [47].

The comparison between sight-allowed and isolated pairs (i.e. pairs composed by octopuses seeing each other or isolated from each other, respectively, during acclimatization) also revealed some intriguing results. Sight-allowed, rather than isolated, pairs showed longer latencies during the cohabitation phase, reaching also higher dominance and exhibiting more numerous avoidances. The explanation of these result is twofold. On the one hand, they suggest that the two octopuses recognise each other as familiar individuals and that such recognition is mediated by sight; on the other, it might be interpreted as a form of habituation to the presence of a conspecific whatever its identity is. For habituation, we mean here a type of learning in which repeated applications of a stimulus to an animal leading to no consequences for it result in decreased responsiveness [55]. Other experiments are thus needed to disentangle the role of the putative sight-mediated IR from habituation in making *O. vulgaris* less aggressive when allowed to interact with a conspecific.

The importance of sight in *O. vulgaris*' social interactions is however revealed when we analyse the behaviour exhibited by familiar pairs, and in particular when we compare sight-allowed and isolated individuals. The former octopuses showed longer latencies and executed more numerous avoidances than isolated individuals. This might be due to the longer time that sight-allowed individuals have been visually exposed to the same conspecific with respect to isolated individuals. In fact, while in isolated pairs the two conspecifics could see each other for 15 min only per day during the 3-d cohabitation phase, sight-allowed octopuses have been kept in visual contact with the same individual for six days in *continuum* (between each cohabitation they were returned to the original tank with transparent partitions). Vision seems to reinforce the effect of physical encounters with a conspecific (as found in many other taxa [56]). The importance of sight in octopuses is thus confirmed. Thanks to their refined eyes [57], [58], cephalopods have an excellent visual ability, rivalling that of higher vertebrates; they use sight to respond to many environmental and biological demands (e.g. predation, navigation, discrimination, learning [28], [59], [60]) and even to communicate with each other, particularly by adopting several body patterns (e.g. passing cloud, zebra crouch, reviewed in [40]).

As also found in *O. bimaculoides* [53] and in other invertebrates (hermit crabs [61] and clawed lobsters [62]), the process of cohabitation between *O. vulgaris* individuals is quick: a 15-min cohabitation is sufficient for an octopus to label the conspecific as familiar and to remember it for at least 1 day. In fact, dominace and avoidances began to reach higher values starting from Day 2 of cohabitation, particularly in isolated pairs. Excellent memory capabilities are well known in octopuses for other cognitive processes: they quickly learn a task [32] and remember it over time, ranging from 5 days for observational learning [32] and 1 week or more for spatial navigation [60] (see [24]). A long memory is certainly advantageous in the case of repeated encounters with the same individual, as was observed in vertebrates (e.g. several months or 1 year in birds: see [10]) and in other invertebrates (e.g. 2 weeks in crayfish [63]; 1–2 weeks in clawed lobsters [62]; 4 days in hermit crabs [61]).

Familiarity between individuals may be achieved through physical contacts, recorded in both the cohabitation and the test phase. Physical contacts were more numerous in unfamiliar pairs (both isolated and sight-allowed) and in isolated pairs, particularly at Day 1, but decreased over time as familiarity between the two individuals increased. The sense of touch, possibly associated with taste [64], seems thus to have a role in binary IR of octopuses as found for other abilities. For example, Boyle [42] suggested that *O. vulgaris* estimates the relative size of a conspecific by the tactile information obtained through Arm Alignement. The importance of the sense of touch for octopuses is confirmed by the large dimension of the subfrontal lobe, the brain region specialized for tactile learning [65].

Another sense that can be involved in the process of familiarization is olfaction. Since visibility is often limited in water, chemical cues are reliable sources of information to aquatic animals even regarding the identity of conspecifics (e.g. in crayfish, lobsters and hermit crabs; see [56]). The potential for chemoreception in octopuses is still understudied (except [66]–[68] and [29]), but there are evidence in the literature that indicate its importance in the life of this taxon. *Octopus vulgaris*, for example, forages through chemotactile exploration [60], detects chemical substances at a distance [69], [70] and, similarly to cuttlefish and squids, uses chemical signals to coordinate its reproductive behaviour [29], [66]. *Sepia officinalis* females seem to rely on chemical cues to select mates [67]. Recently, it has been demonstrated that *Enteroctopus dofleini* learns to open a jar in the presence of chemicals produced by rubbing a herring on it [71].

Of difficult interpretation is the higher threshold for ink jets that we have recorded in isolated rather than in sight-allowed pairs, particularly during the first day of cohabitation, and its decrease during that phase. Typically, ink is used by most cephalopods and by some other molluscs [72] as a means to escape from the attack of either a predator or a conspecific intruder by diverting its attention [68], [73]. Ink also serves as a conspecific alarm substance [68] or as a chemical defence against predators [74]. In our case, the observed decrease in the number of ink jets over time might be due to octopuses being less in danger in the presence of a familiar conspecific. However, we cannot completely discard the hypothesis on the potential use of ink as a social signal, as suggested by Fiorito & Gherardi [75] for *Aplysia fasciata*.

A final intriguing hypothesis that merits to be tested in the near future is that sight, touch and olfaction are part of a multimodal system of information transfer [76], as found in other invertebrates (e.g. the stomatopod *Gonodacytlus festai*, the crayfish *Procambarus clarkii*, the American lobster *Homarus americanus* and the wolf spider *Schizocosa ocreata* [56], [77]–[80]; reviewed in [78]). Indeed, the synchronous use of different media (i.e. multimodality *sensu* [81]) has the clear advantage of improving detection, recognition, discrimination and memorability of signals by the receivers [82], [83].

Further studies are needed to clarify whether *O. vulgaris* is able of true IR –and not simply of binary IR. The role of the different sensory channels involved in this process should be also detailed. Despite the long way ahead to entirely solve the issue, we have shown here, for the first time in cephalopods, that *O. vulgaris* discriminates between familiar and unfamiliar conspecifics. In general, our study has raised new, stimulating questions on the cognitive abilities of this taxon, opening novel scenarios for future comparative research.

## Methods

### Ethics statement

The experiment was carried out in accordance with the Code of Ethics of the World Medical Association (Declaration of Helsinki) for animal experiments, the Proposal for a Directive of the European Parliament and of the Council on the protection of animals used for scientific purposes (Brussels, 5.11.2008), recently passed as bill (Directive 2010/63/EU), and the Uniform Requirements for manuscripts submitted to biomedical journals.

### Subjects, collection, and housing

Sixty individuals of *O. vulgaris* were collected from the Bay of Naples (Italy) and immediately transported to the laboratory of the Stazione Zoologica Anton Dohrn during the summers of 2005 and 2007. Housing followed the standardized protocol as reported in [53]. Immediately upon arrival, each octopus was weighed (range: 114–324 g). Sex was determined at the end of the experiments, through the analysis of the gonads (42 males, 18 females). Octopus pairs were matched by weight.

Twenty four octopus pairs (maximum weight difference: 15%) were isolated in contiguous PVC tanks (maintenance tanks: 65×100×50 cm) covered with a translucent PVC lid to limit animals from escaping. Dark sand was used as substratum at the bottom of each tank and two bricks were placed in a corner to serve as the octopus's den. Tanks were supplied with constant running sea water (38 ppm; depth: 45 cm) at the temperature of 24°C (±0.5°C), under a natural 14:10 h light:dark cycle regime that mimicked the light intensity at 2–6 m sea depth at the latitude of the Bay of Naples. Only individuals in apparently good conditions and without any injury were used for the subsequent experiments.

### Experimental design

**Figure 4:**
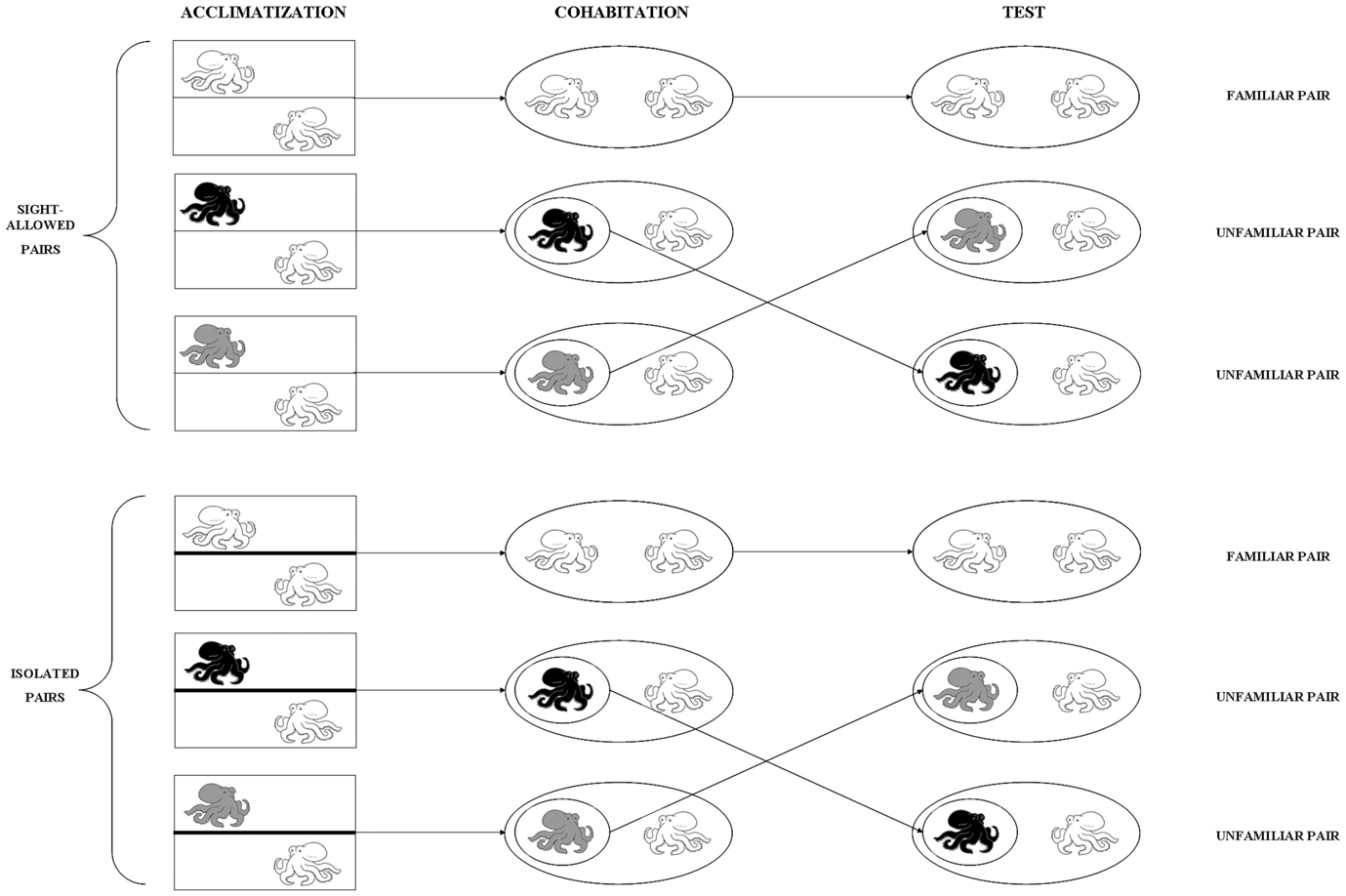
Scheme of the experimental design. Phase 1 (Days 1–3, acclimatization): sight-allowed (and isolated) pairs were maintained in contiguous tanks for three consecutive days separated by a transparent (and opaque) partition that allowed (and blocked) the vision of the conspecific. Phase 2 (Days 4–6, cohabitation): individuals of each pair (both sight-allowed and isolated) were transferred into an experimental tank and were allowed to interact with each other for 15 min every day and for three subsequent days. Phase 3 (Day 7, test): each pair (both sight-allowed and isolated) was subject to either a sham or a real switch; thus, the dominant octopus within the pair encountered either a familiar (in the case of sham switches) or an unfamiliar conspecific (in the case of real switches). Both types of switch were followed by a cohabitation of 15-min.

Experiments were conducted between 1000 h and 1700 h on a weekly basis (from Monday to Sunday) and consisted of three distinct phases, described as follows (see also Fig. 4).

Phase 1 (Days 1–3, acclimatization): octopuses of contiguous tanks were either allowed or not to visually interact with each other. Twelve sight-allowed (and 12 isolated) pairs were maintained in contiguous tanks for three consecutive days separated by a transparent (and opaque) partition that allowed (and blocked) the vision of the conspecific, respectively. In both sight-allowed and isolated pairs, water did not flow between contiguous tanks, excluding the possibility of any exchange of chemical cues. Animals were fed every day in the morning with a live crab (*Carcinus mediterraneus*, mean carapace width: 40 mm). Each octopus was identified by naturally occurring scars and mantle lesions; we avoided the use of tags, numbers or hypodermal ink that could interfere with body patterning.

Phase 2 (Days 4–6, cohabitation): individuals of each pair (from both sight-allowed and isolated pairs) were transferred into an experimental tank (see below) and allowed to interact with each other for 15 min every day for 3 consecutive days. The experimental tank consisted of an ellipsoid opaque PVC container (60×100×50 cm; water depth: 45 cm) with dark sand placed on the bottom, as for the maintenance tanks. Bricks were not provided as den to avoid the octopuses using them as shelter during interactions. Cohabitation was limited to 15 min since preliminary observations had shown that this time in the absence of a den does not cause injury and stress to the animals and is sufficient to establish hierarchies. Here we only analyzed “physical interactions” between the octopuses of each pair and several behavioural categories (see the “Data recorded” section below for details). An interaction began when an octopus approached the conspecific within a few centimetres and ended when one of the individuals retreated at a distance of at least 15 cm. At the start of the cohabitation phase, the experimental tank was divided into two equal compartments separated by an opaque PVC divider with the octopus of each pair occupying a compartment. After 1 min, the experiment started with the removal of the divider. The behaviour of each pair was video-recorded (Sony DCR-TRV33E camera) for subsequent analysis. During the experiment, an experienced observer (ET) recorded the number of interactions and the winner of each interaction; the winner was deemed as the octopus that did not retreat by the end of the interaction. Dominants or alphas (and subordinates or betas) were the octopuses winning more (and less) than 60% of the total interactions executed. Dominance averaged 76%. At the end of the observations, each octopus was returned to its own maintenance tank and fed. The same procedure was adopted for Days 5 and 6.

Phase 3 (Day 7, test): in both sight-allowed and isolated pairs, half of the pairs were subject to a sham switch while the other half to a real switch; thus the dominant octopus within each pair was allowed to encounter either a familiar (in the case of sham switches) or an unfamiliar conspecific (in the case of real switches). Both types of switch were followed by a period of 15-min cohabitation in the experimental tank as Phase 2.

At the end of the experiment (Day 7), octopuses were deeply anaesthetized and sacrificed following [84]. As a result of sex determination, analyses were conducted on 12 sight-allowed pairs (6 female-male and 6 male-male pairs) and 12 isolated pairs (7 female-male and 5 male-male pairs).

### Data recorded

During Phases 2 and 3, other than dominance (defined as the percentage of interactions won by an octopus over the total interactions), we also recorded the following variables:

(1) Latency, in seconds (s), as the time elapsed between the removal of the divider and the first interaction between the two conspecifics.

(2) Number and total length of interactions in seconds (s).

(3) Percentage of avoidance. Avoidance is an interaction without contact between the two conspecifics; individuals approach each other to a distance of a few centimetres but do not enter into physical contact. An octopus either swerves and changes the direction of its movement just before contact or moves away from its resting position to avoid the conspecific.

(4) Five behavioural categories (following, in part [85]): Approach (including following), Withdrawal (retreat and runaway), Incomplete contact (an individual extends an arm, but withdraws it before touching the other), Weak contact (contact is made by extending one or two arms), Strong contact (Arm alignment and Oppose: the arms are applied, sucker to sucker, along their length, in the former, including also part of the web in the latter). For physical contacts we indicate the sum of weak and strong contacts.

(5) Number of ink jets. Octopuses usually jet ink against an intruder when they are in danger [40].

The comparisons between sight-allowed *vs.* isolated pairs and between familiar *vs.* unfamiliar pairs were made by summing, per each pair, the number of behavioural patterns, strong contacts and ink jets separately, as performed by both octopuses in the pair. Pairs (and not individuals) were here taken as sample units for comparison purposes.

### Statistical analyses

The data were tested for normality using the Kolmogorov–Smirnov test and for homogeneity of variance using the Levene test. Percentages were first normalized using the arcsine square root transformation. To correct temporal autocorrelations arising from measurements repeated in time, to prevent temporal pseudoreplication and to control Type I error, a two-way repeated multivariate analysis of variance MANOVA (statistic: Wilk's Lambda *Λ*) was used to compare all the recorded variables between sight-allowed and isolated pairs through time in Phase 2 (factors: days and sight-allowed/isolated pairs). MANOVA was followed by univariate tests for between-subjects effects (statistic: *F*) and then by a *post hoc* Tukey's Honest Significant Differences (HSD). Prior to this test, female-male and male-male pairs within both sight-allowed and isolated pairs were compared to check for possible differences within each of the recorded variables during the cohabitation phase with a two-way repeated measures MANOVA (factors: days and female-male/male-male pairs), followed by univariate tests and Tukey's HDS. If differences were not significant, data from female-male and male-male pairs within both sight-allowed and isolated pairs were merged. A one-way and a two-way MANOVAs, followed by univariate tests, were performed for Phase 2 (factor: sight-allowed/isolated pairs) within each day and for Phase 3 (factors: sight-allowed and isolated pairs, familiar and unfamiliar pairs), respectively. Figures give means ± SE. The level of significance was set at *α* = 0.05.

## Supporting Information

Table S1 Comparisons among the three days of cohabitation (1 = Day 1, 2 = Day 2, 3 = Day 3), and between female-male (fm) and male-male (mm) pairs for the recorded parameters in (A) social (*n* = 12; *n* fm = *n* mm = 6) and (B) isolated pairs (*n* = 12; *n* mf = 7, *n* mm = 5) after a two-way repeated measures MANOVA followed by univariate tests for between-subjects effects (statistic: *F*; factors: days and female-male/male-male pairs), followed by Tukey's HSD. Significant differences are denoted in bold. * means no significant difference after Tukey's HSD.
(DOC)

Table S2 Means and SE of all the analyzed variables for the sight-allowed pairs (SP, *n* = 12) and the isolated pairs (IP, *n* = 12) in the cohabitation phase.
(DOC)

Table S3 P-values of the Tukey's HSD tests following the univariate analyses of Table 1. Significant differences are in bold.
(DOC)

Table S4 Means and SE of all the analyzed variables for the familiar (FA) and unfamiliar pairs (UN) divided in sight-allowed pairs (SP, *n* = 12) and the isolated pairs (IP, *n* = 12) in the test phase.
(DOC)
