# Supplementary material for: I Know My Neighbour: Individual Recognition in Octopus vulgaris
Source: PLoS One. 2011 Apr 13;6(4):e18710. doi: 10.1371/journal.pone.0018710 (PMC3076440; doi:10.1371/journal.pone.0018710)
Supplement: Table S1 — Comparisons among the three days of cohabitation (1 = Day 1, 2 = Day 2, 3 = Day 3), and between female-male (fm) and male-male (mm) pairs for the recorded parameters in (A) social (n = 12; n fm = n mm = 6) and (B) isolated pairs (n = 12; n mf = 7, n mm = 5) after a two-way repeated measures MANOVA followed by univariate tests for between-subjects effects (statistic: F; factors: days and female-male/male-male pairs), followed by Tukey's HSD. Significant differences are denoted in bold. * means no significant difference after Tukey's HSD. (DOC) [file pone.0018710.s001.doc]

|  |  |  |  |  |  |  |  |  |  |  | **A** |
| --- | --- | --- | --- | --- | --- | --- | --- | --- | --- | --- | --- |
|  | DAYS | | | | PAIRS | | | | DAYS x PAIRS | | |
|  | *F* | *df* | *P* | Hierarchy | *F* | *df* | *P* | Hierarchy | *F* | *df* | *P* |
| Latency of first interaction (s) | 1.00 | 2, 33 | 0.38 | 1=2=3 | 1.07 | 1, 34 | 0.31 | fm=mm | 1.15 | 2, 30 | 0.33 |
| Number of interactions | 0.56 | 2, 33 | 0.58 | 1=2=3 | 4.49 | 1, 34 | 0.05 | fm=mm | 1.39 | 2, 30 | 0.27 |
| Length interactions (s) | 0.06 | 2, 33 | 0.95 | 1=2=3 | 0.42 | 1, 34 | 0.52 | fm=mm | 0.93 | 2, 30 | 0.41 |
| Dominance (%) | 0.19 | 2, 33 | 0.68 | 1=2=3 | 1.80 | 1, 34 | 0.21 | fm=mm | 0.79 | 2, 30 | 0.48 |
| Avoidance (%) | 1.98 | 2, 33 | 0.19 | 1=2=3 | 1.60 | 1, 34 | 0.24 | fm=mm | 1.01 | 2, 30 | 0.41 |
| Number of all behavioural patterns | 0.02 | 2, 33 | 0.90 | 1=2=3 | 0.09 | 1, 34 | 0.77 | fm=mm | 0.02 | 2, 30 | 0.98 |
| Physical contacts (%) | 0.71 | 2, 33 | 0.42 | 1=2=3 | 1.35 | 1, 34 | 0.28 | fm=mm | 1.05 | 2, 30 | 0.39 |
| Number of ink jets | 0.79 | 2, 33 | 0.39 | 1=2=3 | 0.78 | 1, 34 | 0.38 | fm=mm | 0.80 | 2, 30 | 0.40 |
|  |  |  |  |  |  |  |  |  |  |  |  |
|  |  |  |  |  |  |  |  |  |  |  |  |
|  |  |  |  |  |  |  |  |  |  |  | **B** |
|  | DAYS | | | | PAIRS | | | | DAYS x PAIRS | | |
|  | *F* | *df* | *P* | Hierarchy | *F* | *df* | *P* | Hierarchy | *F* | *df* | *P* |
| Latency of first interaction (s) | 5.68 | 2, 33 | **0.04** | 1=2=3* | 0.48 | 1, 34 | 0.49 | fm=mm | 1.15 | 2, 30 | 0.11 |
| Number of interactions | 0.70 | 2, 33 | 0.50 | 1=2=3 | 0.20 | 1, 34 | 0.66 | fm=mm | 0.50 | 2, 30 | 0.61 |
| Length interactions (s) | 1.32 | 2, 33 | 0.28 | 1=2=3 | 0.05 | 1, 34 | 0.83 | fm=mm | 0.26 | 2, 30 | 0.78 |
| Dominance (%) | 10.29 | 2, 33 | **0.008** | 2=3>1 | 6.84 | 1, 34 | **0.03** | fm>mm | 4.90 | 2, 30 | **0.04** |
| Avoidance (%) | 5.67 | 2, 33 | **0.04** | 1=2=3* | 0.12 | 1, 34 | 0.74 | fm=mm | 0.29 | 2, 30 | 0.76 |
| Number of all behavioural patterns | 0.16 | 2, 33 | 0.70 | 1=2=3 | 0.26 | 1, 34 | 0.62 | fm=mm | 0.17 | 2, 30 | 0.84 |
| Physical contacts (%) | 6.80 | 2, 33 | **0.02** | 1>2=3 | 0.27 | 1, 34 | 0.62 | fm=mm | 0.36 | 2, 30 | 0.71 |
| Number of ink jets | 9.75 | 2, 33 | **0.01** | 1>2=3 | 1.67 | 1, 34 | 0.23 | fm=mm | 0.60 | 2, 30 | 0.57 |
