# Supplementary material for: I Know My Neighbour: Individual Recognition in Octopus vulgaris
Source: PLoS One. 2011 Apr 13;6(4):e18710. doi: 10.1371/journal.pone.0018710 (PMC3076440; doi:10.1371/journal.pone.0018710)
Supplement: Table S2 — Means and SE of all the analyzed variables for the sight-allowed pairs (SP, n = 12) and the isolated pairs (IP, n = 12) in the cohabitation phase. (DOC) [file pone.0018710.s002.doc]

|  |  | **Cohabitation phase** | | |
| --- | --- | --- | --- | --- |
| **Variable** | **Pair** | Day 1 | Day 2 | Day 3 |
| Latency of first interaction (s) | SP | 54.91± 23.31 | 73.36 ± 29.50 | 94.55 ± 33.44 |
|  | IP | 7.23 ± 2.27 | 33.15 ± 4.57 | 38.31 ± 4.30 |
| Number of interactions | SP | 10.17± 3.82 | 9.75± 3.07 | 10.17± 3.84 |
|  | IP | 10.33 ± 4.75 | 10.75 ± 4.79 | 10.33 ± 4.77 |
| Length interactions (s) | SP | 146.75 ± 41.40 | 148.33 ± 30.26 | 157.92 ± 26.94 |
|  | IP | 224.08 ± 28.62 | 197.15 ± 34.72 | 190.54 ± 33.79 |
| Dominance (%) | SP | 73.17 ± 4.31 | 77.53 ± 4.83 | 80.43 ± 2.62 |
|  | IP | 51.08 ± 2.79 | 73.54 ± 11.19 | 77.93 ± 11.43 |
| Avoidance (%) | SP | 52.09 ± 6.07 | 57.77 ± 7.90 | 55.53 ± 5.52 |
|  | IP | 37.04 ± 5.28 | 51.85 ± 5.61 | 50.34 ± 9.13 |
| Number of all behavioural patterns | SP | 62.25 ± 10.10 | 56.58 ± 8.46 | 52.00 ± 7.08 |
|  | IP | 63.67 ± 10.87 | 61.33 ± 7.28 | 55.92 ± 6.16 |
| Physical contacts (%) | SP | 22.63 ± 2.75 | 20.83 ± 4.76 | 20.98 ± 2.71 |
|  | IP | 40.52 ± 2.63 | 29.51 ± 4.56 | 28.22 ± 3.94 |
| Number of ink jets | SP | 0.92 ± 0.38 | 0 | 0 |
|  | IP | 2.75 ± 0.83 | 0.25 ± 0.13 | 0.09 ± 0.08 |
