# Supplementary material for: I Know My Neighbour: Individual Recognition in Octopus vulgaris
Source: PLoS One. 2011 Apr 13;6(4):e18710. doi: 10.1371/journal.pone.0018710 (PMC3076440; doi:10.1371/journal.pone.0018710)
Supplement: Table S3 — P-values of the Tukey's HSD tests following the univariate analyses of Table 1. Significant differences are in bold. (DOC) [file pone.0018710.s003.doc]

|  | **Cohabitation phase** | | |
| --- | --- | --- | --- |
| **Variable** | Day 1 *vs* Day 2 | Day 1 *vs* Day 3 | Day 2 *vs* Day 3 |
| Latency of first interaction (s) | 0.49 | 0.17 | 0.77 |
| Number of interactions | 0.97 | 0.74 | 0.86 |
| Length interactions (s) | 0.99 | 1.00 | 1.00 |
| Dominance (%) | **0.005** | **<0.001** | 0.42 |
| Avoidance (%) | 0.05 | 0.05 | 0.95 |
| Number of all behavioural patterns | 0.60 | 1.00 | 0.56 |
| Physical contacts (%) | **0.02** | **0.02** | 0.99 |
| Number of ink jets | **<0.001** | **<0.001** | 0.99 |
