# Supplementary material for: I Know My Neighbour: Individual Recognition in Octopus vulgaris
Source: PLoS One. 2011 Apr 13;6(4):e18710. doi: 10.1371/journal.pone.0018710 (PMC3076440; doi:10.1371/journal.pone.0018710)
Supplement: Table S4 — Means and SE of all the analyzed variables for the familiar (FA) and unfamiliar pairs (UN) divided in sight-allowed pairs (SP, n = 12) and the isolated pairs (IP, n = 12) in the test phase. (DOC) [file pone.0018710.s004.doc]

|  |  | **Test phase** | |
| --- | --- | --- | --- |
| **Variable** | **Pair** | SP | IP |
| Latency of first interaction (s) | FA | 73.83 ± 15.13 | 33.33 ± 0.81 |
|  | UN | 13.33 ± 8.95 | 11.17 ± 1.54 |
| Number of interactions | FA | 10.33 ± 1.33 | 10.75 ± 0.79 |
|  | UN | 11.33 ± 1.25 | 12.33 ± 1.07 |
| Length interactions (s) | FA | 138.50 ± 24.58 | 143.17 ± 26.64 |
|  | UN | 250.33 ± 38.17 | 284.00 ± 49.86 |
| Dominance (%) | FA | 82.67 ± 5.54 | 80.17 ± 2.40 |
|  | UN | 63.00 ± 6.93 | 63.00 ± 4.29 |
| Avoidance (%) | FA | 64.40 ± 4.14 | 49.44 ± 4.29 |
|  | UN | 34.11 ± 6.27 | 31.60 ± 7.48 |
| Number of all behavioural patterns | FA | 53.50 ± 8.37 | 56.17 ± 14.23 |
|  | UN | 56.17 ± 5.76 | 58.33 ± 4.65 |
| Physical contacts (%) | FA | 20.29 ± 4.47 | 21.11 ± 1.76 |
|  | UN | 42.15 ± 7.47 | 42.21 ± 7.90 |
